# ITRAQ-based proteomics analysis of human ectopic endometrial stromal cells treated by Maqian essential oil

**DOI:** 10.1186/s12906-023-04246-8

**Published:** 2023-11-27

**Authors:** Liu-yang Zhang, Ting-ting Huang, Li-ping Li, Dan-ping Liu, Yong Luo, Wan Lu, Ning Huang, Peng-peng Ma, Yan-qiu Liu, Ping Zhang, Bi-cheng Yang

**Affiliations:** 1 Jiangxi Key Laboratory of Birth Defect Prevention and Control, Jiangxi Maternal and Child Health Hospital, Nanchang, China; 2 Center for Integrative Conservation & Yunnan Key Laboratory for Conservation of Tropical Rainforests and Asian Elephants, Xishuangbanna Tropical Botanical Garden, Chinese Academy of Sciences, Mengla, 666303 Yunnan China

**Keywords:** Chinese herbal medicine, Endometriosis, Proteome, Essential oil

## Abstract

**Background:**

Endometriosis is a common and complex syndrome characterized by the presence of endometrial-like tissue outside the uterus. Chinese medicine has been recently found to show good efficacy in treating endometriosis. Our previous results revealed that Maqian fruit essential oil (MQEO) could inhibit the proliferation and induce apoptosis of ectopic endometrial stromal cells (EESCs), but the mechanisms remain unclear. In this study, we aim to explore the molecular mechanism of MQEO’s specific effects in EESCs.

**Methods:**

We conducted a quantitative proteomics analysis by iTRAQ on EESCs treated with MQEO or DMSO. Then deep analysis was performed based on differentially expressed proteins, including Gene Ontology enrichment analysis, pathway enrichment analysis and protein interaction analysis. Candidate protein targets were subsequently verified by western blotting.

**Results:**

Among 6575 identified proteins, 435 proteins exhibited altered expression levels in MQEO-treated EESCs. Of these proteins, most were distributed in signal transduction as well as immune system and the most significantly altered pathway was complement and coagulation cascades. Moreover, two differentially expressed proteins (Heme oxygenase 1 and Acyl-CoA 6-desaturase) were verified and they can be potential biomarkers for endometriosis treatment.

**Conclusions:**

Our proteomic analysis revealed distinct protein expression patterns induced by MQEO treatment in EESCs, highlighting the potential of MQEO for endometriosis treatment and biomarker discovery.

**Supplementary Information:**

The online version contains supplementary material available at 10.1186/s12906-023-04246-8.

## Background

Endometriosis is estimated to affect 10% of reproductive-age women [1], with symptoms such as painful periods, chronic pelvic pain, infertility, painful sex, pain on defecation and urination, etc. Despite of its unclear pathogenesis, genetic and genomic, hormonal, angiogenic, neurological, and immunological factors are all found to be implicated in endometriosis [2]. Nowadays, the mainstream treatments of endometriosis include surgery, medication and the combination of the two. Surgery often results in relapse due to the difficulty in removing multiple organs involved in endometriosis. Many medical treatments have unwanted side effects and the regular hormonal therapy limit fertility. Consequently, new therapies which can relief symptoms while preserving fertility are urgently needed. In the past decade, Chinese herbal medicine and ethnic medicine were found to have good efficacy in endometriosis (such as alleviating dysmenorrhea, shrinking ovarian endometriotic cysts and promoting pregnancy) [3] with fewer side effects and lower recurrence rates after drug withdrawal compared with traditional hormonal therapy [4, 5].

Endemic to China Xishuangbanna Dai Autonomous Prefecture, *Zanthoxylum myriacanthum var. pubescens* Huang, also known as Maqian, is distinguished by its villous rachises, petiolules and leaves. With special lemon fragrance of its leaves and fruits, Maqian is widely consumed as a cooking spice by Dai people. Meanwhile, it is also a traditional Dai herb for treating insect bite, swelling and pain, and gastrointestinal disorders [6]. The Maqian fruit essential oil (MQEO) is rich in limonene (67.06%) and shows strong antimicrobial and anti-inflammatory activities in LPS-stimulated macrophages and THP-1 cells [6, 7]. Oral administration of MQEO also showed protective effect in DSS-induced colitis in mice by attenuating MPO and MMP-9 expression and proinflammatory cytokine mRNAs in colon tissue [7]_._ Furthermore, our previous study showed that MQEO inhibited proliferation and induced apoptosis of human ectopic endometrial stromal cells in a dose-and time-dependent manner [8]. However, the exact molecular targets of MQEO in endometrial stromal cells remain unknown. In recent years, with the development and application of omics technology, the pathogenesis of endometriosis has been well studied [9, 10]. As we all know, genetic central dogma is one of the most important and fundamental rules in modern biology, and proteins play key roles in almost all cell functions. Thus, we utilized the isobaric tags for the relative and absolute quantitation (iTRAQ) method, one of the mature proteomic analysis techniques, to investigate potential molecular targets of MQEO in endometrial stromal cells.

## Materials and methods

### Cell culture and treatment

The primary ectopic endometrial stromal cells (EESCs) used in this study were derived from patients with ovarian endometriosis who were histologically confirmed in Jiangxi Provincial Maternal and Child Health Hospital (Nanchang, China) [11] and were extracted as previously described [12, 13]. After subculture, a part of cells were frozen in a liquid nitrogen tank for ultra-low temperature preservation waiting for next resuscitation. Resuscitated cells were cultured in DMEM/F12 medium containing 10% fetal bovine serum, 100 IU/mL penicillin and 0.1 mg/mL streptomycin. The essential oil from Maqian (MQEO) was provided by professor Zhang Ping, researcher at Xishuangbanna Tropical Botanical Garden, Chinese Academy of Sciences. The chemical composition of MQEO was similar to previously reported [7]. Freshly isolated EESCs were divided into experimental group and control group, which were treated with 0.075% MQEO (v/v) or 0.075% dimethyl sulfoxide (DMSO) for 48 h, respectively. All treatments were repeated in triplicate.

### Protein extraction and proteolysis

Cells were washed twice in PBS after removal of growth media, centrifuged at 1000* g* for 5 min to collect the cells, the cell pellets were lysed with RIPA lysis buffer (Beyotime, China) containing a protease inhibitor cocktail (Sigma-Aldrich, USA), incubated at 4 ℃ for 2 h. The cell debris was removed by centrifugation at 12,000 rpm at 4 ℃ for 15 min and the supernatant was the protein solution. The quality control was performed with the method of Bradford quantification and SDS-PAGE. The protein samples were diluted with 0.5 M TEAB beforehand to make the final concentration of urea lower than 2 M and SDS less than 0.1%. Then the protein samples were mixed with trypsin according to a ratio of 1:20, vortexed and centrifuged at low speed for 1 min, then incubated at 37℃ for 4 h. The digested peptide liquid was removed for desalting and freeze-drying.

### Peptide labeling and fractionation

An aliquot of 50μL isopropanol was mixed with room temperature iTRAQ labeling reagent. Peptide samples were dissolved in 0.5 M TEAB and added to the corresponding iTRAQ labeling reagent. Different iTRAQ labels were employed for different sample peptides. The labeled peptides were then left to stand at room temperature for 2 h. The Shimadzu LC-20AB liquid phase system was used, and the separation column was a 5um 4.6 × 250 mm Gemini C18 column for liquid phase separation of the sample. The dried peptide samples were reconstituted with mobile phase A (5% ACN pH 9.8) and injected, eluting at a flow rate of 1 mL/min by following gradients: 5% mobile phase B (95% ACN, pH 9.8) for 10 min, 5% to 35% mobile phase B for 40 min, 35% to 95% mobile phase B for 1 min, mobile phase B for 3 min, and 5% mobile phase B for 10 min. The elution peak was monitored at a wavelength of 214 nm and one component was collected per minute, and the samples were combined according to the chromatographic elution peak map to obtain 20 fractions, which were then freeze-dried.

### HPLC and mass spectrometry detection

The dried peptide samples were reconstituted with mobile phase A (2% ACN, 0.1% FA), centrifuged at 20,000 g for 10 min, and the supernatant was taken for injection. Separation was performed by Thermo UltiMate 3000 UHPLC. The sample was first enriched in trap column and desalted, and then entered a self-packed C18 column (75 μm internal diameter, 3 μm column size, 25 cm column length) and separated at a flow rate of 300nL/min by the following effective gradient: 0 ~ 5 min, 5% mobile phase B (98% ACN, 0.1% FA); 5 ~ 45 min, mobile phase B linearly increased from 5 to 25%; 45 ~ 50 min, mobile phase B increased from 25 to 35%; 50 ~ 52 min, mobile phase B rose from 35 to 80%; 52 ~ 54 min, 80% mobile phase B; 54 ~ 60 min, 5% mobile phase B. The nanoliter liquid phase separation end was directly connected to the mass spectrometer. The peptides separated by liquid phase chromatography were ionized by a nanoESI source and then passed to a tandem mass spectrometer Q-Exactive HF (Thermo Fisher Scientific, San Jose, CA) for DDA (Data Dependent Acquisition) mode detection. The main parameters were set: ion source voltage was set to 1.9 kV, MS1 scanning range was 350 ~ 1,500 m/z; resolution was set to 60,000; MS2 starting m/z was fixed at 100; resolution was 15,000. The ion screening conditions for MS2 fragmentation: charge 2 + to 6 + , and the top 20 parent ions with the peak intensity exceeding 10,000. The ion fragmentation mode was HCD, and the fragment ions were detected in Orbitrap. The dynamic exclusion time was set to 30 s. The AGC was set to: MS1 3E6, MS2 1E5.

### Protein identification and quantitation

The raw MS/MS data was converted into MGF format and then searched by the protein identification software Mascot through alignment in corresponding databases. In the meantime, quality control was performed to determine if a reanalysis step was needed. Next, the qualified data must pass a certain screening threshold to obtain the final credible protein identification results. Later, *IQuant*, an automated software independently developed by Beijing Genomics institution (BGI), was applied to quantify protein levels. Differentially expressed proteins were selected from the quantitative results. Further, we performed deep analysis based on differentially expressed proteins, including Gene Ontology (GO) enrichment analysis, Kyoto Encyclopedia of Genes and Genomes (KEGG) pathway enrichment analysis [14] and protein interaction analysis. All the procedures above were based on a false discovery rate (FDR) of no more than 1%.

### Western blotting

Protein samples extracted from EESCs were processed by SDS-PAGE and transferred to the nitrocellulose membrane. The membrane was then blocked with PBS containing 5% skimmed milk power and incubated with primary antibodies at 4 °C overnight. Later, the membrane was incubated with appropriate secondary IgG antibody for 1 h at room temperature. The result was examined by ECL chemiluminescence. Densitometry values were normalized to β-actin or GAPDH.

### Statistical analysis

Measurement data were presented in the form of “the means ± standard deviations”. With SPSS (version 19.0), statistical analysis for multiple group comparisons was performed by oneway ANOVA. The difference between two groups was analyzed by Student’s t test. *P* < 0.05 was regarded as statistically significant.

## Results

### Protein identification and quantitation results

**Fig. 1:**
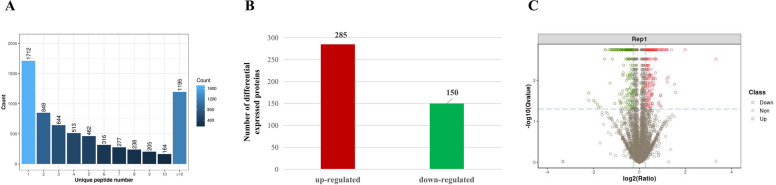
Identification and quantitation proteomic analysis results. **A** Unique peptide number distribution. The X-axis indicates the unique peptide number of each protein and the Y-axis indicates the corresponding protein number. Most of the identified proteins contain less than 10 peptides and protein quantity becomes less with the increase in peptide number. **B** Number of significantly differential expressed proteins. Totally 435 proteins were defined to be significantly different, among which 285 were up-regulated and 150 were down-regulated. **C** Volcano plot of log2 fold-change (X-axis) versus -log10 Qvalue (Y-axis, indicating the probability that the protein is differentially expressed). Red dots represent proteins significantly up-regulated, green for significantly down-regulated, and gray for no-significant change

Based on the standard of 1% FDR, 44,716 peptides and 6575 proteins were identified in total (Fig. 1 A). For these 6575 proteins, analysis of significant difference between DMSO and MQEQ treated groups was performed. Proteins of significant difference were screened as fold change > 1.2 and Q-value < 0.05. According to this criterion, 435 proteins were defined to be differentially expressed proteins (DEPs), among which 285 were up-regulated and 150 were down-regulated (Fig. 1B and C). The top 20 up-regulated and down-regulated proteins are listed separately in Tables 1 and 2.

**Table 1:** List of the top 20 up-regulated proteins in MQEQ-treated EESCs

Accession	Protein name	Fold change	*P* value
Q14147	Probable ATP-dependent RNA helicase DHX34	3.347755422	0.03866958
Q7Z5H4	Vomeronasal type-1 receptor 5	2.844688021	0.01062212
P52823	Stanniocalcin-1	2.673433739	0.000512193
P08254	Stromelysin-1	2.639501666	0.0181334
O95500	Claudin-14	2.392929207	0.01584208
P02748	Complement component C9	2.358412795	0.005135254
A6NK44	Glyoxalase domain-containing protein 5	2.352060279	0.006570461
P02771	Alpha-fetoprotein	2.273137612	0.001340501
Q99988	Growth/differentiation factor 15	2.268304555	0.000291106
P02741	C-reactive protein	2.254483861	0.01495653
Q8WXE9	Stonin-2	2.239639991	0.03200757
Q8NET8	Transient receptor potential cation channel subfamily V member 3	2.153177863	0.000571239
P01031	Complement C5	2.149578598	0.000935405
P09601	Heme oxygenase 1	2.143340453	7.36E-06
P36575	Arrestin-C	2.136414407	0.007485843
P10643	Complement component C7	2.12290508	0.001495639
Q8WTS1	1-acylglycerol-3-phosphate O-acyltransferase ABHD5	2.102369241	0.000321776
Q9UKQ9	Kallikrein-9	2.095386419	0.0105733
Q03181	Peroxisome proliferator-activated receptor delta	2.077167019	0.001605747
Q0VG06	Fanconi anemia core complex-associated protein 100	2.074085963	0.000219121

**Table 2:** List of the top 20 down-regulated proteins in MQEQ-treated EESCs

Accession	Protein name	Fold change	*P* value
Q86Y22	Collagen alpha-1(XXIII) chain	0.358118393	0.00015303
P45452	Collagenase 3	0.56253854	0.02214124
O95864	Acyl-CoA 6-desaturase	0.573638277	6.12E-05
Q9Y5W5	Wnt inhibitory factor 1	0.579178503	0.003537251
Q9H2H9	Sodium-coupled neutral amino acid transporter 1	0.587552607	0.002869714
P11388	DNA topoisomerase 2-alpha	0.603146716	0.0124694
Q9NPB9	Atypical chemokine receptor 4	0.641899105	0.01359628
P02452	Collagen alpha-1(I) chain	0.645907938	0.00014629
O00767	Acyl-CoA desaturase	0.646179524	0.001044735
O14867	Transcription regulator protein BACH1	0.649429437	0.006773781
Q53QV2	Protein LBH	0.65004797	0.00046562
Q13322	Growth factor receptor-bound protein 10	0.657127039	0.001246585
Q6ZU67	BEN domain-containing protein 4	0.660648194	0.000941751
Q96MH7	Uncharacterized protein C5orf34	0.66755777	0.005640629
Q8N2N9	Ankyrin repeat domain-containing protein 36B	0.671461157	0.03894651
Q71RG4	Transmembrane and ubiquitin-like domain-containing protein 2	0.675864553	0.000881633
P0DH78	RING finger protein 224	0.678190184	0.03047435
Q96JB3	Hypermethylated in cancer 2 protein	0.68153325	0.003271633
O00418	Eukaryotic elongation factor 2 kinase	0.693631423	0.003123135
Q29980	MHC class I polypeptide-related sequence B	0.694639615	0.0133043

### GO enrichment analysis of DEPs

**Fig. 2:**
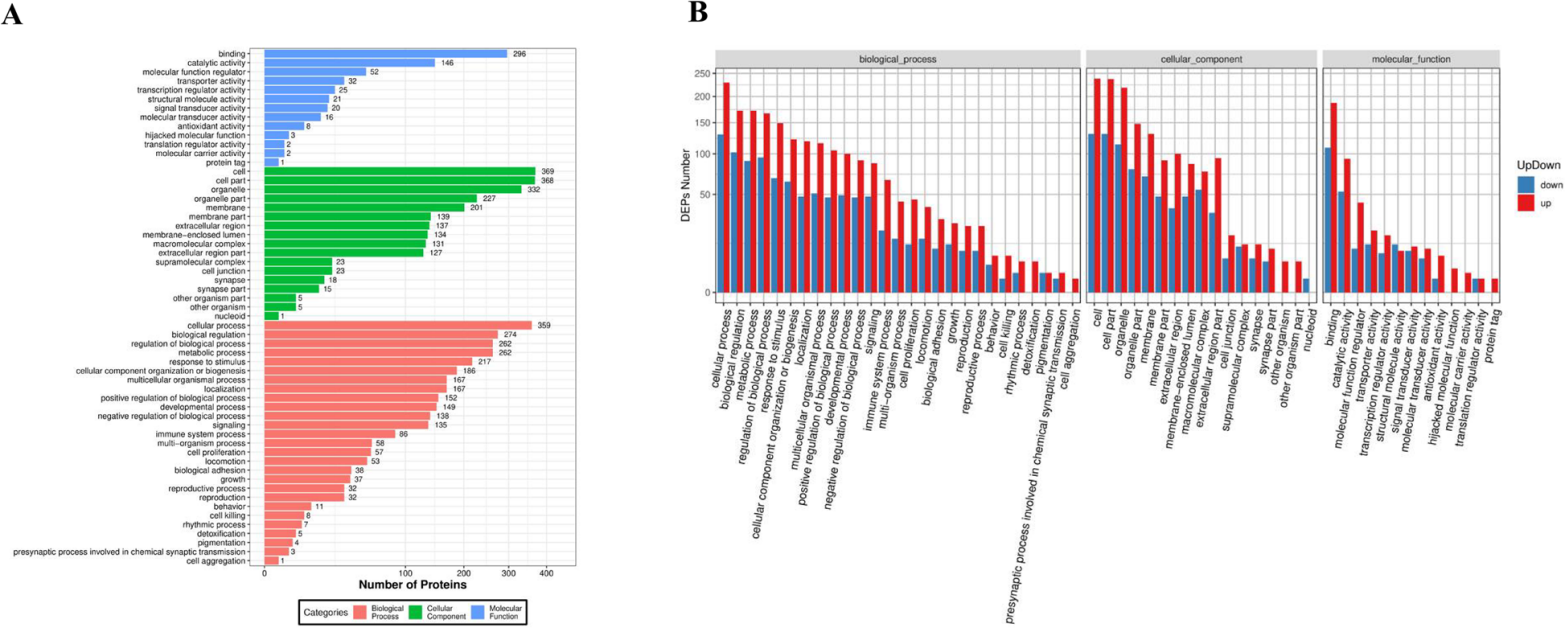
GO analysis of DEPs. **A** GO classification of DEPs. The X-axis indicates DEP protein count, and the Y-axis indicates GO term. **B** GO classification of up/down-regulated DEPs. The X-axis indicates GO term, and the Y-axis indicates up/down-regulated protein count

GO enrichment analysis shows the important or typical biology functions in measured samples. Molecular function analysis results showed that the majority of identified proteins were relevant to binding. Cell, cell part and organelle were the top 3 in cellular component. As for biological process, most proteins were involved in cellular process. In almost all GO items of DEPs, the number of MQEO up-regulated proteins was much more than that of down-regulated (Fig. 2).

### KEGG pathway enrichment analysis of DEPs

**Fig. 3:**
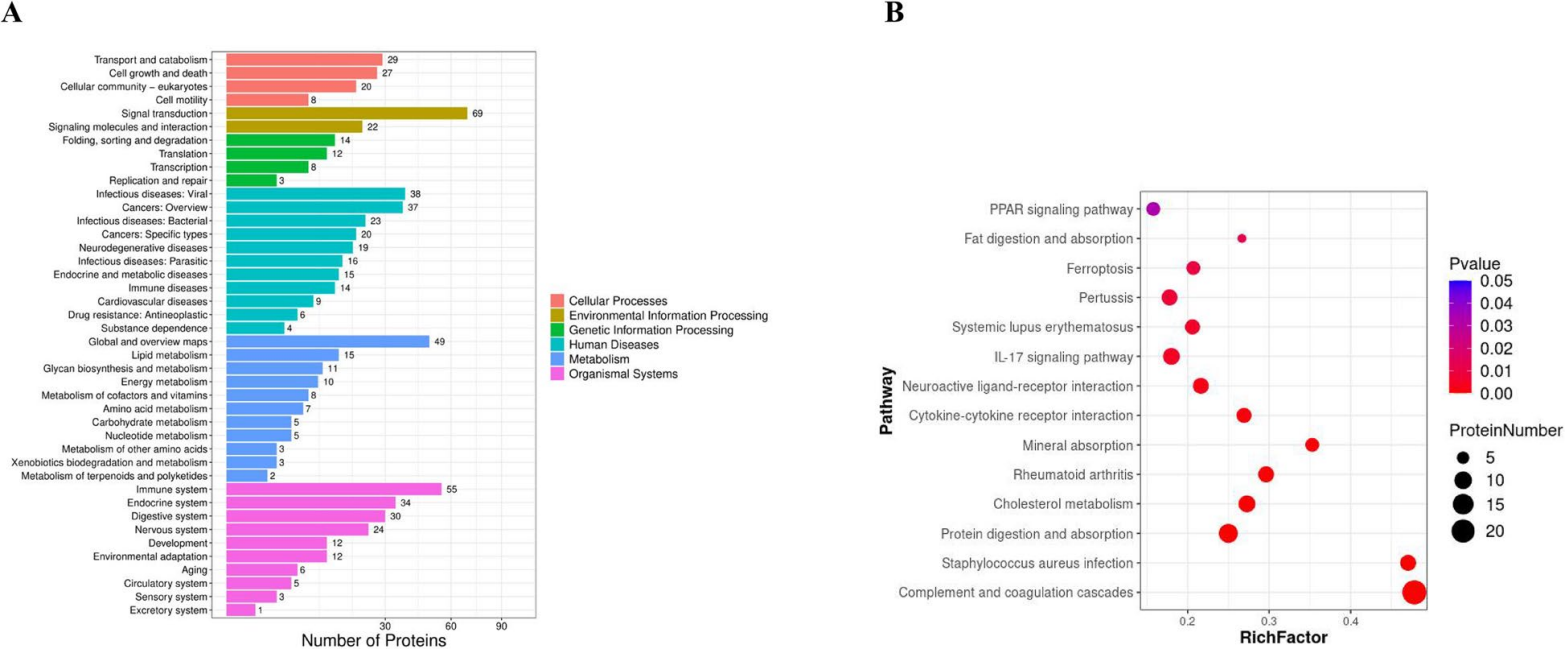
Pathway analysis of DEPs. A Pathway classification of DEPs. X-axis indicates DEP number, Y-axis indicates pathway name. B Statistics of pathway enrichment of DEPs in each pairwise. Rich factor is the ratio of DEP number annotated in this pathway term to all protein number annotated in this pathway term. Greater rich factor means greater intensiveness. *P* value ranges from 0 ~ 1, and less *P* value means greater intensiveness

KEGG pathway enrichment analysis showed that MQEO-induced DEPs were involved in a wide range of pathways (Fig. 3A), whereas only 14 pathways were with significant changes (*P* < 0.05) (Fig. 3B). The top 10 significantly altered pathways were as follows: complement and coagulation cascades (ko04610); staphylococcus aureus infection (ko05150); protein digestion and absorption (ko04974); cholesterol metabolism (ko04979); rheumatoid arthritis (ko05323); mineral absorption (ko04978); cytokine-cytokine receptor interaction (ko04060); neuroactive ligand-receptor interaction (ko04080); IL-17 signaling pathway (ko04657); systemic lupus erythematosus (ko05322).

### Protein–protein interaction (PPI) of DEPs

**Fig. 4:**
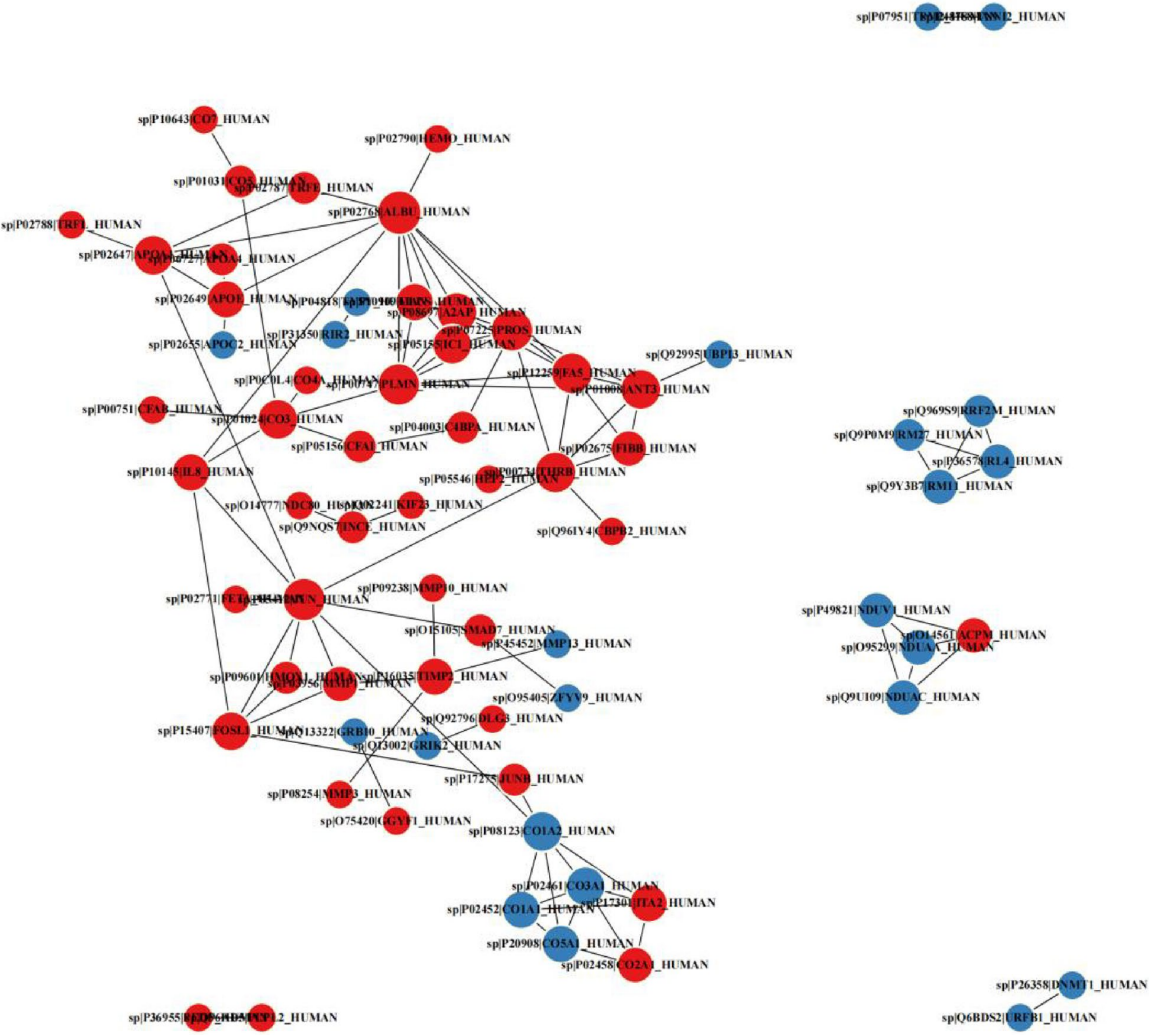
PPI Network of DEPs. Red nodes represent up-regulated proteins; blue nodes represent down-regulated proteins

In general, proteins interact with each other to form complexes that perform their respective functions. The interactions include direct (physical) and indirect (functional) associations. They stem from computational prediction, from knowledge transfer between organisms, and from interactions aggregated from other (primary) databases. As shown in Fig. 4, PPI of MQEO induced DEPs is a complex network.

### Verification of iTRAQ results by western blotting

**Fig. 5:**
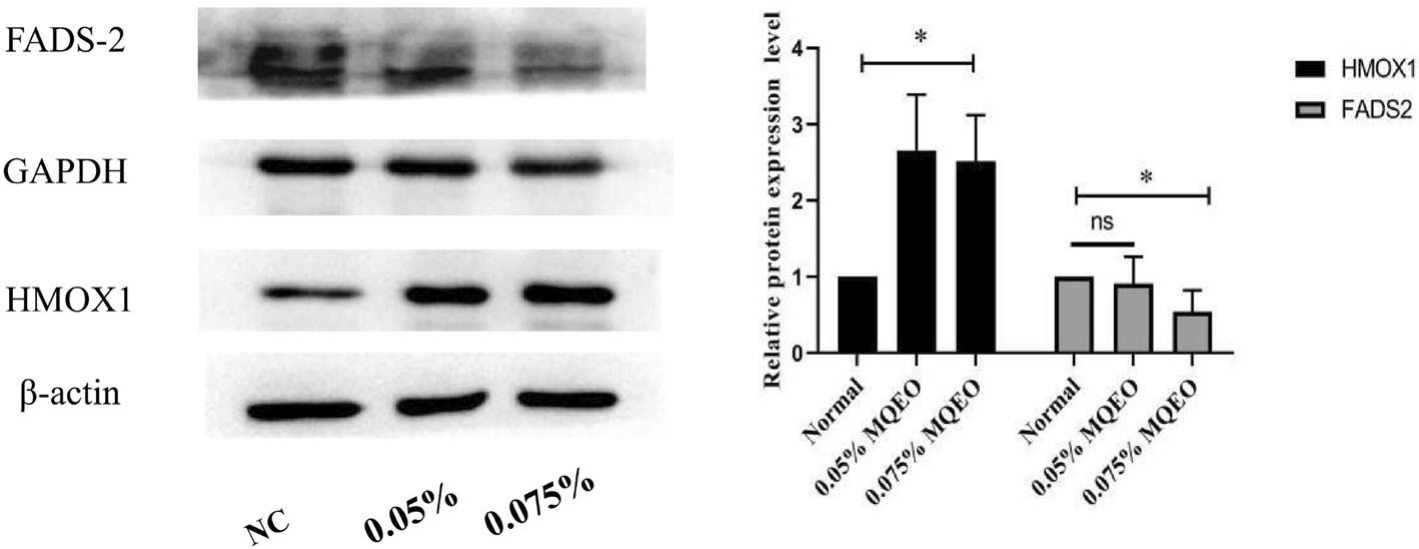
Expression level of representative DEPs analyzed by western blotting. Proteins HMOX1 is upregulated and protein FADS-2 is downregulated. β-actin or GAPDH was used as the loading control. *P < 0.05 compared with the control group

From Tables 1 and 2, we know that Heme oxygenase 1 (HMOX1) is significantly up-regulated (fold change 2.143340453, *P* value 7.36E-06) and Acyl-CoA 6-desaturase (fatty acid desaturase 2, FADS-2) is significantly down-regulated (fold change 0.646179524, *P* value 6.12E-05) in MQEO-treated EESCs. Consistent with iTRAQ results, compared with the control group, the western blotting results (Fig. 5) also showed that MQEO increased HMOX1 protein expression more than twofold and MQEO significantly decreased FADS-2 at 0.075% (v/v) concentration. Therefore, altered protein expression levels induced by MQEO in EESCs were confirmed by western blotting.

## Discussion

Compared with modern medicine, ethnic medicine has certain advantages: being environmentally friendly, effective in chronic and complex diseases, with fewer side effects, thus providing new ideas for drug development [15–21]. MQEO is extracted by hydrodistillation of dried Maqian fruit and has been shown to have very strong antimicrobial, anti-oxidant, anti-inflammatory, and anti-diabetic effect [6, 7]. Gas chromatography-mass spectrometry (GC–MS) analysis result reveals that MQEO consists of 16 volatile compounds with limonene as the principal constituent [9]. It has been widely reported that limonene has a number of therapeutic effects, including antimicrobial [22, 23], anti-inflammatory [24, 25], anti-tumor [26, 27], antioxidant [28, 29], neuroprotective and gastroprotective [30, 31]. MQEO exhibited much stronger anti-inflammatory effect than d-limonene alone [6], indicating that other major and minor ingredients in MQEO are also important for MQEO’s biological effects.

In our previous research, it was found that MQEO dosage-dependently reduced the viability, motility and migration of EESCs. But the exact molecular mechanism remains unclear. Therefore, in this study we applied iTRAQ, a powerful proteomic-based method for biomarker screening, to explore MQEO’s effect on EESCs. As shown in the results, 44,716 peptides and 6575 proteins were identified in total, among which 435 DEPs were identified in the MQEO-treated group in comparison to the control group according to our criteria. Further analysis showed that 285 DEPs were up-regulated and 150 were down-regulated. These results showed that there are significant differences between MQEO-treated EESCs and the control, which may be candidate therapeutic targets of MQEO in treating endometriosis.

Furthermore, GO enrichment analysis of DEPs revealed that binding and cellular process were the most highlighted molecular function and biological process respectively. These biological functions are closely associated with the process of endometriosis. Besides, KEGG pathway analysis and PPI of DEPs showed a complex network, in which most DEPs were distributed in signal transduction as well as immune system with complement and coagulation cascades as the most significantly altered pathways. Our result is consistent with reported interaction between the complement system and the coagulation cascade in the development of endometriosis. Multi-omics analysis has revealed that up-regulated expression of complement (C1S, C1QA, C1R and C3) was positively correlated with tissue factor in endometriosis [32]. Endometriosis was considered as a chronic inflammatory and immune dysfunctional disease with the characteristics of ectopic endometrial tissue implantation and growth [33, 34]. The complement system, involved in inflammation and autoimmune disease, was one of the indispensable immune mechanisms in endometriosis [35]. Complement system plays an important role in innate immunity through chemotaxis, immune-complex elimination, formation of membrane attack complex and cell lysis. The formation of thrombus in coagulation cascades is somehow similar to complement cascades [36]. Markiewski [37] found that complement and coagulation systems work together as partners in response to inflammatory. According to Markus and Amara [38, 39], thrombin substituted for the C3-dependent C5 convertase in the absence of C3; while in the presence of C3, thrombin did also generate C3a dose- and time-dependently. Briefly, the interaction between complement and coagulation cascade is of great clinical significance, since disruption of it may result in diseases.

Meanwhile, we performed a literature analysis of DEPs identified by iTRAQ and then verified by western blotting (Fig. 5). We found that HMOX1 and FADS-2 were significantly up-regulated and down-regulated respectively in MQEO-treated EESCs compared with DMSO treated control, which may provide more details about the mechanism of MQEO in endometrial stromal cells. Promoting the degradation of heme into carbon monoxide (CO), iron (Fe2 +) and biliverdin, HMOX1 is an important anti-oxidative enzyme [40]. It is reported that HMOX1 is highly expressed in endometriosis [41, 42] and gene analysis confirms that HMOX1 gene polymorphism is associated with endometriosis [43]. FADS-2, a member of fatty acid desaturase, is rarely studied in endometriosis, but both FADS-2 and HMOX1 are involved in ferroptosis, a new programmed cell death characterized by the accumulation of lipid reactive oxygen species (ROS) and dependence of iron [44]. There are several intracellular pathways regulating ferroptosis, one of which is the *p62-Keap1-Nrf2-HMOX1* pathway [45]. Cellular stress activates *p62* by its phosphorylation and stimulates the interaction between *p62* and Kelch-like ECH-associated protein 1 (*Keap1*), resulting in disassociation of nuclear factor-E2-related factor 2 (*Nrf2*) from the *p62/Keap1* complex. Then the free and phosphorylation-activated *Nrf2* translocates to the nucleus and induces HMOX1 gene expression. As the result, HMOX1 degrades heme and releases Fe2 + , which impairs iron homeostasis and triggers ferroptosis. *Zeyu Wang* proved that knockdown of HMOX1 could reduce the sensitivity of cells to ferroptosis [46]. Consequently, HMOX1 was found to be a promoter of ferroptosis [47, 48]. On the contrary, knockdown of FADS2 decreases the ferroptosis-associated negative regulators at the mRNA level and increases the iron levels and lipid [49]. As a result, FADS-2 was regarded as a rate-limiting factor in ferroptosis [49, 50]. Considering that endometriosis is characterized by ferroptosis resistance [51–53] and MQEO is able to up-regulate *Nrf2* [54], we propose that MQEO may active ferroptosis through up-regulating *Nrf2-HMOX1* to achieve inhibition of EESCs. These may become potential endometriosis molecular treatment targets but more in-depth studies on the ferroptosis mechanisms underlying the functions of MQEO in EESCs are still warranted.

## Conclusion

In conclusion, we applied an iTRAQ-based proteomic method to investigate the potential mechanisms of MQEO’s effect in EESCs. The proteomic results revealed changed expression levels of a number of proteins involved in inflammatory and metabolic pathways. And the significantly differential expressed proteins were verified by western blotting. We identified HMOX1 and FADS-2 as potential endometriosis molecular treatment targets in the future. Taken together, our results shed a new insight into the therapeutic intervention of endometriosis.

### Supplementary Information

**Additional file 1: Suppleme figure 1.** Expression level of HMOX1 is upregulated in EESC1 after MQEO pretreated. **Suppleme figure 2.** Expression level of FADS2 is downregulated in EESC1 after MQEO pretreated. **Suppleme figure 3.** Expression level of HMOX1 is upregulated in EESC2 and EESC3 after MQEO pretreated. **Suppleme figure 4.** Expression level of FADS2 is downregulated in EESC2 and EESC3 after MQEO pretreated.

## Data Availability

The datasets generated and analyzed during the current study are available from the corresponding author on reasonable request.

## Abbreviations

MQEO: Maqian fruit essential oil
EESCs: Ectopic endometrial stromal cells
DMSO: Dimethyl sulfoxide
DEPs: Differentially expressed proteins
HMOX1: Heme oxygenase 1
FADS-2: Fatty acid desaturase 2
