# Supplementary material for: ITRAQ-based proteomics analysis of human ectopic endometrial stromal cells treated by Maqian essential oil
Source: BMC Complement Med Ther. 2023 Nov 27;23:427. doi: 10.1186/s12906-023-04246-8 (PMC10680300; doi:10.1186/s12906-023-04246-8)
Supplement: Supplementary file 1 — Additional file 1: Suppleme figure 1. Expression level of HMOX1 is upregulated in EESC1 after MQEO pretreated. Suppleme figure 2. Expression level of FADS2 is downregulated in EESC1 after MQEO pretreated. Suppleme figure 3. Expression level of HMOX1 is upregulated in EESC2 and EESC3 after MQEO pretreated. Suppleme figure 4. Expression level of FADS2 is downregulated in EESC2 and EESC3 after MQEO pretreated. [file 12906_2023_4246_MOESM1_ESM.pdf]

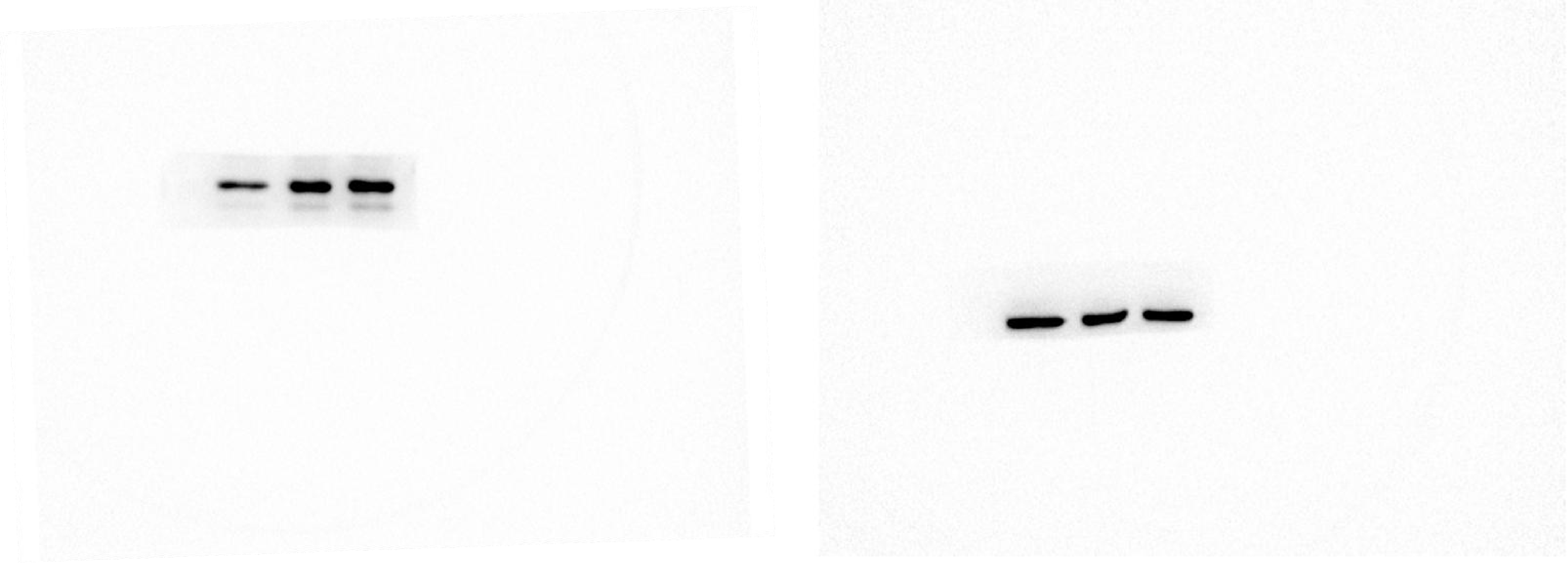

HMOX1 EESC1 0.05%MQEO 0.075%MQEO

ACTB EESC1 0.05%MQEO 0.075%MQEO

suppleme figure 1：Expression level of HMOX1 is upregulated in EESC1 after MQEO pretreated.

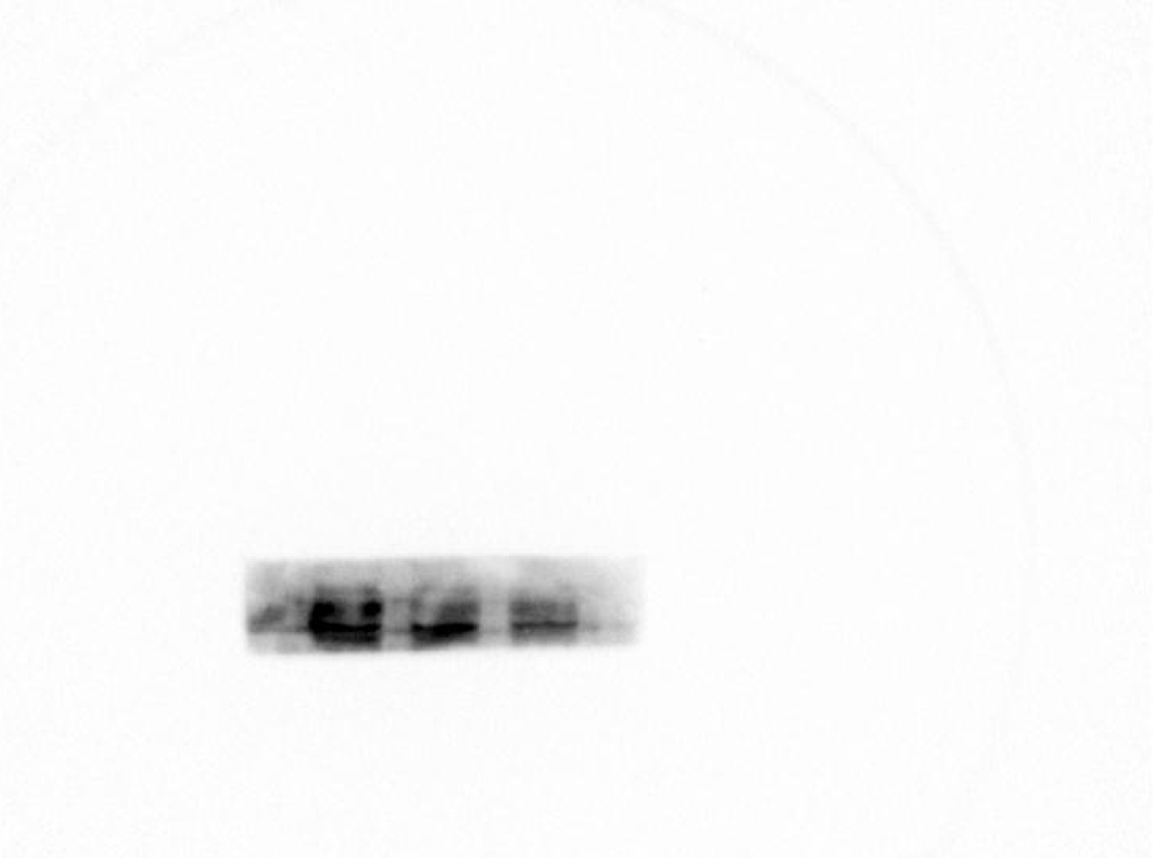

FADS2 EESC1 0.05%MQEO 0.075%MQEO

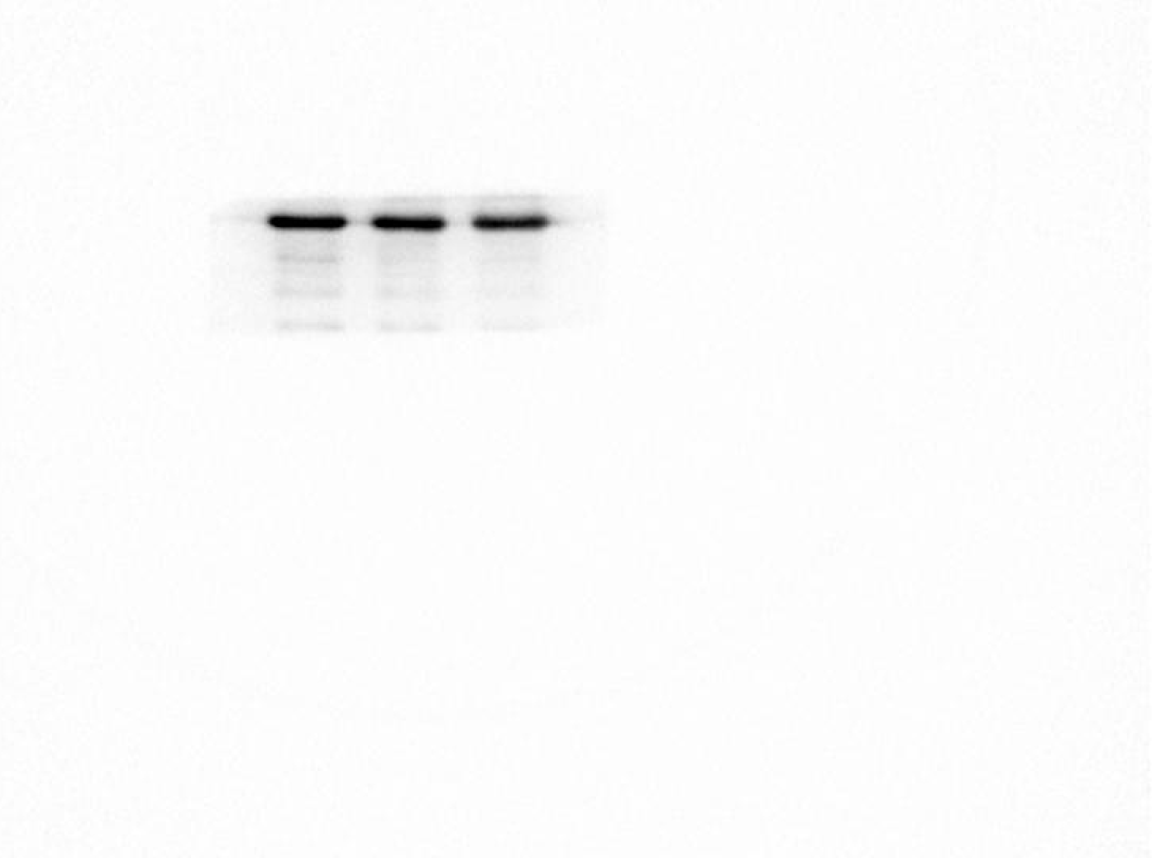

GAPDH EESC1 0.05%MQEO 0.075%MQEO

suppleme figure 2：Expression level of FADS2 is downregulated in EESC1 after MQEO pretreated.

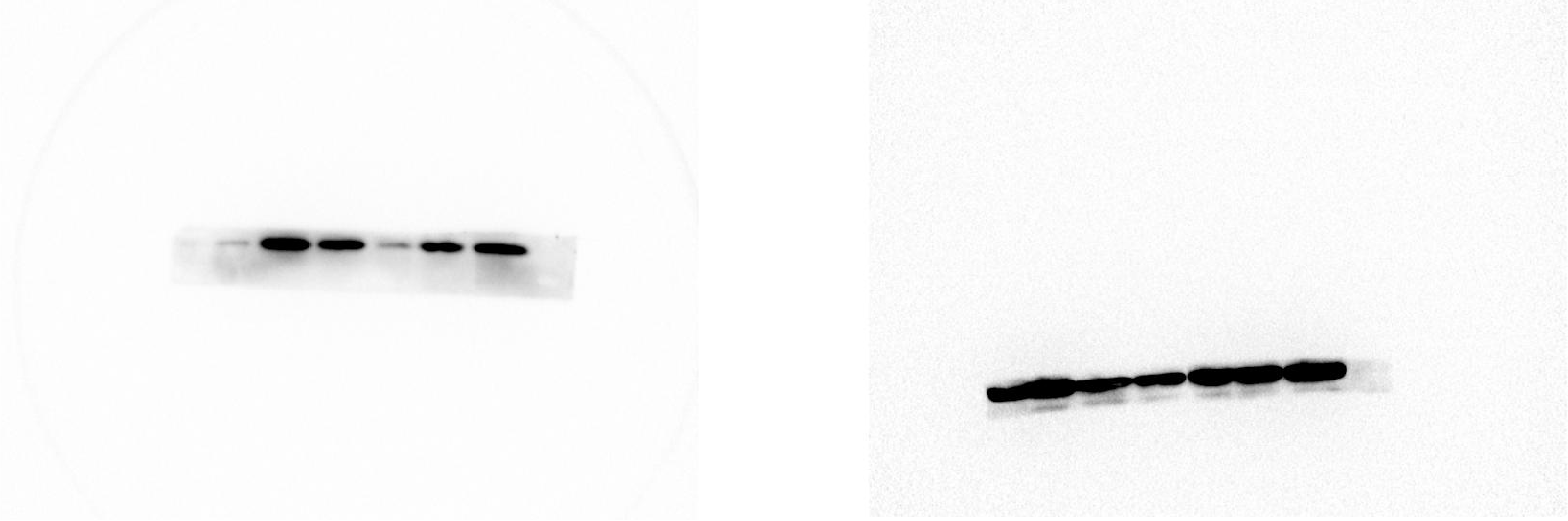

ACTB

HMOX1

EESC2 0.05%MQEO 0.075%MQEO EESC3 0.05%MQEO 0.075%MQEO

EESC2 0.05%MQEO 0.075%MQEO EESC3 0.05%MQEO 0.075%MQEO

suppleme figure 3：Expression level of HMOX1 is upregulated in EESC2 and EESC3 after MQEO pretreated.

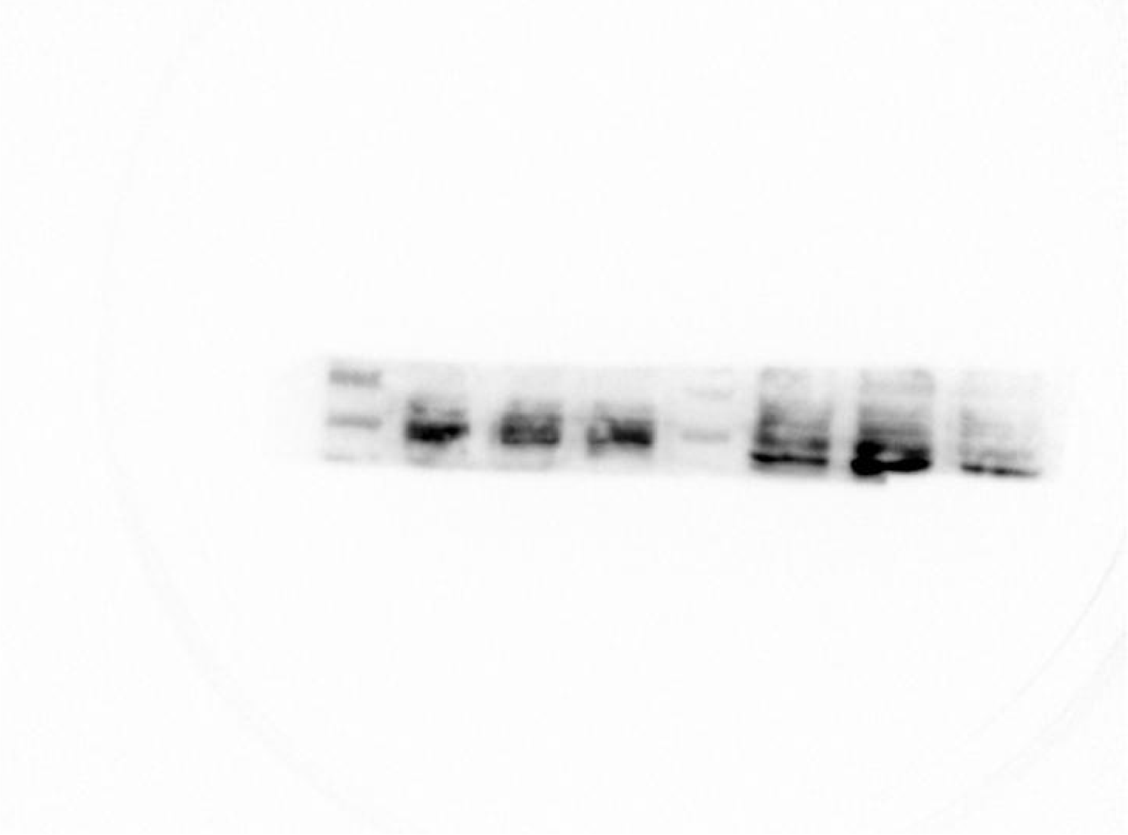

FADS2

EESC2 0.05%MQEO 0.075%MQEO EESC3 0.05%MQEO 0.075%MQEO

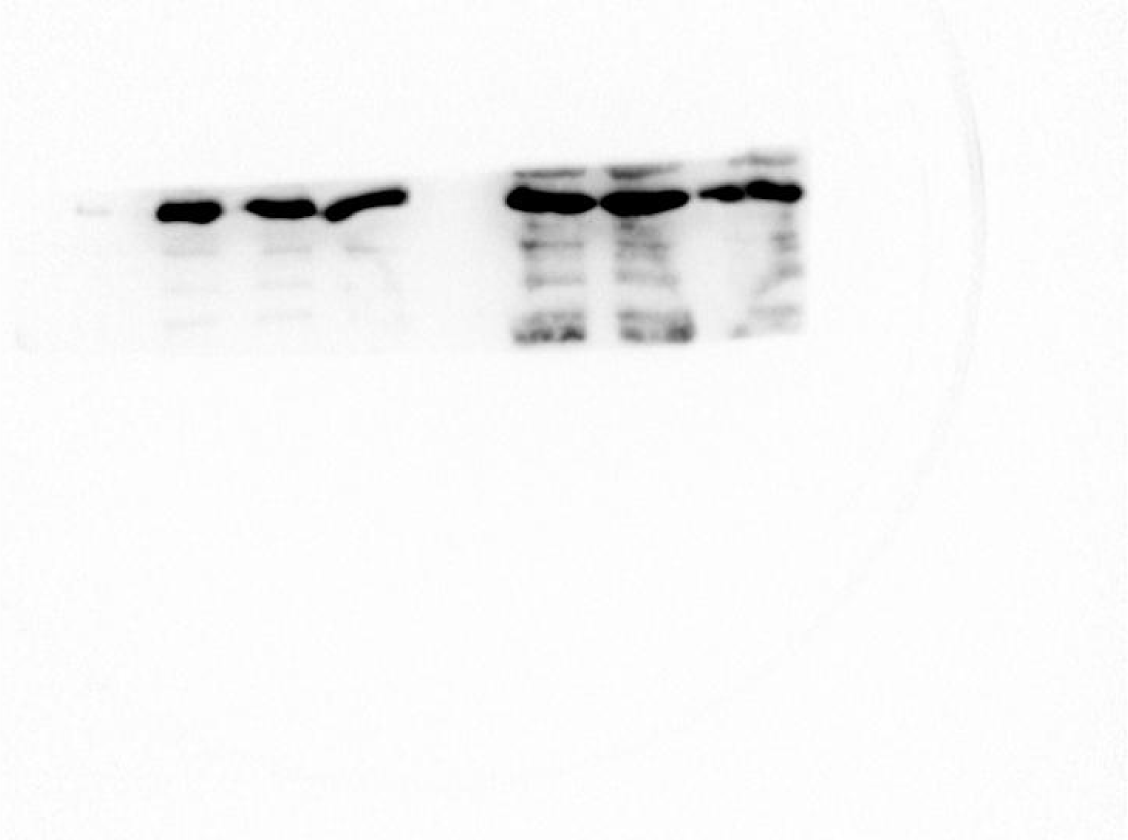

GAPDH

EESC2 0.05%MQEO 0.075%MQEO EESC3 0.05%MQEO 0.075%MQEO

suppleme figure 4：Expression level of FADS2 is downregulated in EESC2 and EESC3 after MQEO pretreated.
